# Timing of autologous stem cell transplantation from last chemotherapy affects lymphocyte collection and survival in non-Hodgkin lymphoma

**DOI:** 10.1111/j.1365-2141.2006.06088.x

**Published:** 2006-06

**Authors:** Shernan G Holtan, Luis F Porrata, David J Inwards, Stephen M Ansell, Ivana N Micallef, Patrick B Johnston, Mark R Litzow, Dennis A Gastineau, Svetomir N Markovic

**Affiliations:** 1 Division of Internal Medicine/Department of Medicine, Mayo Clinic College of MedicineRochester, MN, USA; 2 Division of Hematology/Department of Medicine, Mayo Clinic College of MedicineRochester, MN, USA

**Keywords:** chemotherapy, absolute lymphocyte count, non-Hodgkin lymphoma, autologous stem cell transplantation, survival

## Abstract

Autograft absolute lymphocyte count (A-ALC) is a prognostic factor for survival in non-Hodgkin lymphoma (NHL) after autologous stem cell transplantation (ASCT). An A-ALC is dependent upon the preaphaeresis absolute lymphocyte count (PA-ALC) at the time of aphaeresis. It was hypothesised that the time interval from last chemotherapy (TILC) to aphaeresis affects PA-ALC. One hundred and sixty consecutive NHL patients who underwent ASCT at the Mayo Clinic between 1996 and 2001 were evaluated. A strong correlation between TILC and PA-ALC (*r* = 0.67, *P* < 0.0001) was identified. Higher PA-ALC was observed in TILC ≥55 d compared with TILC <55 d [median: 7.0 vs. 3.8 × 10^9^/l], *P* < 0.0001). TILC as a continuous variable was identified as a prognostic factor for overall survival (OS) [hazard ratio (HR) = 0.989, *P* < 0.01] and progression-free survival (PFS) (HR = 0.992, *P* < 0.0492). Median OS and PFS were longer in the TILC ≥55 d vs. TILC <55 d group (not reached vs. 21 months, *P* < 0.0008; 76 vs. 9 months, *P* < 0.0025, respectively). Multivariate analysis demonstrated TILC to be an independent prognostic indicator for OS and PFS. These findings suggest that the immune status of the host at the time of aphaeresis may predict survival after ASCT.

The infused autograft absolute lymphocyte count (A-ALC) is an independent prognostic factor for survival in non-Hodgkin lymphoma (NHL) status after autologous stem cell transplantation (ASCT) (Porrata *et al*, 2004). An A-ALC is dependent on the preaphaeresis absolute lymphocyte count (PA-ALC) at the time of aphaeresis (Porrata *et al*, 2004). The factors affecting PA-ALC, as a surrogate marker of the host immune status at the time of aphaeresis, remain unknown. This study evaluated the hypothesis that PA-ALC is directly dependent upon the time interval from last chemotherapy (TILC), as a marker of immune recovery time from the myelosuppressive effects of chemotherapy prior to proceeding with aphaeresis collection.

## Patients and methods

### Patient population

From 1996 to 2001, 160 consecutive NHL patients underwent autologous peripheral blood stem cell transplantation at our institution. All patients were included in this retrospective study, in which the data were prospectively collected over time and entered into a computerised database. Response to therapy, relapse and survival, data were updated continuously. No patients were lost to follow-up. All patients gave written, informed consent allowing the use of their medical records for medical research. Approval of the study was obtained from the Mayo Clinic Institutional Review Board and was in accordance with US Federal regulations and the Declaration of Helsinki.

### Objectives of the study

The primary objective of the study was to assess the correlation between TILC and PA-ALC. The secondary objective was to determine the impact on overall survival (OS) and progression-free survival (PFS) based on TILC. The REMARK (REporting recommendations for tumour MARKer prognostic studies) guidelines (McShane *et al*, 2005) were used to report TILC as a prognostic factor for survival. The PA-ALC was calculated from the complete blood cell count (CBC) performed prior to each aphaeresis collection. The absolute white blood cell count (WBC) from the autograft was calculated as follows: autograft bag volume (ml) × autograft WBC cells/ml (×10^9^ cells/l) × 0.001. The autograft WBC was obtained using the ACT 10 Series Analyzer Coulter, Miami, FL, USA and the percentage of lymphocytes was determined microscopically using Wright stain. The A-ALC for each collection was calculated as follows: [(total absolute WBC) × (% Lymphocytes)]/kg. Absolute lymphocyte count at day 15 (ALC-15) after ASCT was obtained from the CBC at day 15 post-ASCT.

### Prognostic factors

The international age-adjusted prognostic index [age (≥60 vs. <60 years), lactate dehydrogenase (LDH) >normal for age/sex, performance status (PS) (≥2 vs. <2), extranodal sites (≥2 vs. <2) and stage (I/II vs. III/IV)] at the time of transplantation, in addition to clinical response status before transplantation, were used in the study.

### Peripheral blood stem cell collection

Patients received granulocyte-colony stimulating-factor (10 *μ*g/kg/d) for five to seven consecutive days by subcutaneous injection. Patients underwent daily aphaeresis collections until a target of 2.0 × 10^6^CD34 cells/kg or greater was achieved. There were no purged or CD34-selected stem cells collections.

### Conditioning regimens

In total, 90 patients received BEAM [BCNU (carmustine; 300 mg/m^2^), etoposide (100 mg/m^2^), cytarabine (ARA-C) (100 mg/m^2^) and melphalan (140 mg/m^2^)], 62 patients received BEAC [BCNU (140 mg/m^2^), etoposide (100 mg/m^2^), ARA-C (100 mg/m^2^) and cyclophosphamide (35 mg/kg)] and eight patients received cyclophosphamide (60 mg/m^2^) and total body irradiation (TBI) (12 Gy).

### Response and survival

Response criteria were based on the guidelines from the NHL International Workshop (Cheson *et al*, 1999). Complete response (CR) was defined as complete regression of all measurable or evaluable disease including radiologically demonstrable disease, bone marrow involvement or peripheral blood involvement. Partial (PR) was defined as a reduction in the sum of the products of measurable lesions’ longest diameter and perpendicular diameters of 75% or greater, with a 50% or greater decrease in hepatomegaly or splenomegaly (measured from the costal margin), if there was previous known liver or spleen involvement. Stable disease was defined as less than PR, but was not progressive disease. Disease progression was defined as a 50% or more increase in the sum of the products of the longest diameter and the perpendicular diameter of measurable lesion (S) from the prestudy measurement, the appearance of new lesions, or a 2-cm increase in spleen or liver size because of lymphoma. Relapsed disease was defined as the appearance of any new lesion or increase by 50% or more in the size of previously involved sites.

Overall survival was measured from the date of transplantation to the date of death or last follow-up. PFS was defined as the time from transplantation to disease progression, relapse, death or last follow-up. Those who died were considered to have had disease progression unless documented evidence clearly indicated that no progression had occurred.

### Statistical analysis

Overall survival and PFS were analysed using the approach of Kaplan and Meier (1958). Differences between survival curves were tested for statistical significance using the two-tailed log-rank test. TILC was assessed as a continuous variable and dichotomised based on finding the optimal cut-off point based on the log-rank statistic (Crowley & McCoy, 1993). Cox proportional hazards model (Cox, 1972) was used to evaluate TILC as a prognostic factor for post-transplant OS and PFS times as well as to assess and adjust for other known prognostic factors. Risk ratios reported are for risks associated with patients having a TILC ≥55 d vs. TILC <55 d. Other prognostic factors tested included age (≥60 vs. <60 years), LDH >normal for age/sex, PS (≥2 vs. <2), extranodal sites (≥2 vs. <2), stage (I/II vs. III/IV), CR status at transplantation and international prognostic index (IPI) ≥2.

In addition to the evaluation of TILC and its prognostic significance for OS and PFS, its utility as a marker for PA-ALC (≥5 × 10^9^/l) was also assessed. This cut-off and definition of PA-ALC was based on our previous publication. The choice of optimal cut-off of TILC was based on its utility as a marker of PA-ALC using box plot, receiver-operator characteristic (ROC) curves, and area under the curve (AUC) analyses as well as its prognostic value for post-transplant OS. Chi squared tests were used to determine relationships between categorical variables; the Wilcoxon's rank-sum tests were used to determine associations between categorical and continuous variables, and Spearman's correlation coefficients were used to evaluate associations for continuous variables. All *P*-values represented were two-sided, and statistical significance was declared at *P* < 0.05.

## Results

### Patient characteristics

The median age at the time of transplantation for the study cohort was 54 years (range: 23–73). Distributions of additional baseline characteristics for these patients are presented in Table I and are summarised according to TILC <55 d vs. ≥55 d. No differences between the groups were identified for patient characteristics or prognostic factors. CHOP (cyclophosphamide, hydroxydaunomycin, oncovin and prednisone) followed by DHAP (dexamethasone, high-dose ARA-C and platinol) was the most frequent (31% of the cases) chemotherapy combination used prior to ASCT. Other regimens used included ESHAP (etoposide, Solu-Medrol, ARA-C and platinol), fludarabine, ICE (ifosfamide, carboplatin and etoposide), MINE (mesna, ifosfamide, novantrone and etoposide), promace-cytobom (prednisone, methotrexate, adriamycin, cyclophosphamide, etoposide, cytarabine, bleomycin, oncovin and methotrexate) and VANDERBILT. We identified no association between the chemotherapy regimens and TILC as a continuous variable (*P* = 0.25) or TILC ≥55 d as a dichotomised variable (*P* = 0.31).

**Table I:** Baseline characteristics of patients according to the time interval from last chemotherapy (TILC) to peripheral blood absolute lymphocyte count at the time of stem cell mobilisation and collection.

Characteristics	TILC <55 d (*n* = 89)	TILC ≥55 d (*n* = 71)	*P*-value
Age(years)			
Median, range	52 (23–73)	54 (31–72)	0.96
Gender			
Female	31	27	0.74
Male	58	44	
Histology			
Diffuse large cell	59	45	0.10
Mantle cell	6	9	
Follicular	12	15	
T cell	9	1	
Other	3	1	
Prognostic factors at time of autologous stem cell transplantation (ASCT)			
Age			
≥60 years	29	21	0.73
LDH			
>Normal	30	20	0.49
Performance status			
<2	84	68	0.68
Extranodal sites			
<2	84	69	0.46
Stage			
III/IV	63	45	0.40
International prognostic index			
0	19	16	0.62
1	35	30	
2	23	20	
3	12	5	
Number of pretransplant regimens			
1	14	10	0.45
2	53	44	
3	19	11	
4	3	6	
Clinical outcome pre-ASCT			
Complete remission	10	12	0.50
Partial remission	74	56	
First relapse	1	1	
Second relapse	2	0	
Refractory	2	2	
Conditioning regimens			
BEAC	33	29	0.45
BEAM	53	37	
CTX/TBI	3	5	

BEAC = BCNU, etoposide, cytarabine, and cyclophosphamide; BEAM = BCNU, etoposide, cytarabine, and melphalan; CTX/TBI, cyclophosphamide/total body irradiation; LDH, lactate dehydrogenase.

The median follow-up was 34.5 months (range: 1–145 months). At the time of these analyses, 84 patients (53%) had died. Seventy-six patients died of lymphoma. In the TILC <55 d group, two patients died of acute respiratory distress syndrome, one patient of pneumonia, one patient of acute myeloid leukemia and one patient of chronic myeloid leukemia. In the TILC ≥55 d group, two patients died of acute myeloid leukemia and one patients of renal failure. None of the patients developed clinically evident autologous graft-*versus*-host-disease.

### Role of TILC on PA-ALC

In an attempt to identify factors that influence PA-ALC, we assessed the utility of TILC, as a marker of immune recovery time from the myelosuppressive effects of chemotherapy prior to proceeding with aphaeresis collection. Box plot analysis showed that the median TILC was significantly higher in those patients with a PA-ALC ≥5 × 10^9^/l vs. PA-ALC <5 × 10^9^/l at the time of aphaeresis collection (70 d vs. 43 d respectively *P* < 0.0001). An ROC and sensitivity/specificity curves and AUC analyses showed that TILC was a significant marker for PA-ALC (AUC = 0.86, *P* < 0.0001). Based on these results, as well as evaluating various TILC cut-off points as prognostic factors for OS, TILC ≥55 d was considered optimal. Therefore, this cut-off point was evaluated for TILC in all subsequent analyses in this study.

**Fig. 1:**
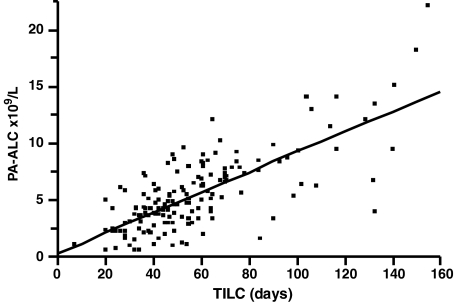
Scatterplot comparing time interval from last chemotherapy (TILC) (days) and preaphaeresis absolute lymphocyte count (PA-ALC) (×10^9^/l). A strong positive correlation was identified between TILC and PA-ALC. (Spearman's correlation rho factor, *r* = 0.67, *P* < 0.0001).

With regard to the association between TILC and PA-ALC, these dichotomised variables were found to be significantly correlated with each other (*P* < 0.0001) as were their continuous counterparts (*r*_s_ = 0.67, *P* < 0.0001) (Fig 1). No other patient characteristic or prognostic factor was found to be associated with PA-ALC. A strong association was found between PA-ALC and A-ALC (*r*_s_ = 0.73, *P* < 0.0001) and between A-ALC and ALC-15 (*r*_s_ = 0.67, *P* < 0.0001), as reported before (Porrata *et al*, 2004). Because of the strong associations between PA-ALC with A-ALC and A-ALC with ALC-15, when these values were compared between the TILC groups, higher values were found in the TILC ≥55 d compared with TILC <55 d (Table II).

**Table II:** Comparison of PA-ALC/A-ALC/ALC-15 between TILC groups.

Factors	TILC <55 d	TILC ≥55 d	*P*-values
PA-ALC, median (range)	3.8 (0.56–9.53)	7.0 (1.55–22.1)	<0.0001
A-ALC, median (range)	0.38 (0.41–1.44)	0.65 (0.04–2.21)	<0.0001
ALC-15, median (range)	0.35 (0.07–1.06)	0.56 (0.04–6.99)	<0.0001

ALC-15, absolute lymphocyte count at day 15 postautologous stem cell transplantation (×10^9^/l); A-ALC, autograft absolute lymphocyte count (×10^6^/kg); PA-ALC, preaphaeresis absolute lymphocyte count (×10^9^/l).

### Post-transplant survival and TILC

**Fig. 2:**
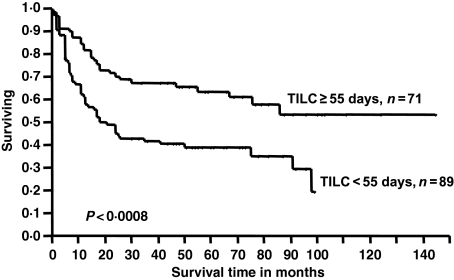
Overall survival of patients with TILC ≥55 d vs. patients with a TILC <55 d. The median OS was not reached in the TILC ≥55 d group and was 21 months in the TILC <55 d group. The OS rates at 5 years were 64% and 39%, respectively (*P* < 0.0008).

**Fig. 3:**
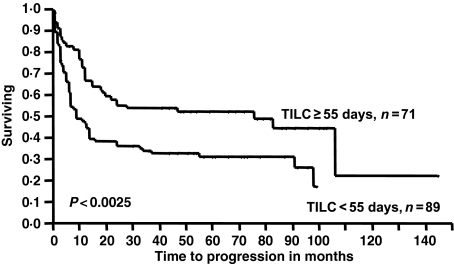
Progression-free survival (PFS) of patients with TILC ≥55 d vs. patients with a TILC <55 d. The median PFS was 76 months in the TILC ≥55 d group and 9 months in the TILC <55 d group. The OS rates at five years were 52% and 31%, respectively (*P* < 0.0025).

The median post-transplant OS (Fig 2) and PFS (Fig 3) times were significantly better for patients with a TILC ≥55 d compared with patients with a TILC <55 d (not reached vs. 21 months, *P* < 0.0008; not reached vs. 9 months, *P* < 0.0025, respectively). Significant factors in the univariate analysis for OS included TILC (as a continuous and dichotomised variable), LDH, PS and IPI (Table III). In the multivariate analysis (Table IV), TILC remained a significant factor when compared with LDH, PS and IPI. Similarly, univariate analysis showed that TILC (as a continuous and dichotomised variable) was a significant factor for PFS, in addition to the IPI (Table III). Multivariate analyses showed the TILC remained a significant factor when compared with the IPI (Table IV). Given the strong correlation and agreement between TILC and PA-ALC/A-ALC/ALC-15, only TILC was included in the multivariate model.

**Table III:** Univariate analysis for overall and PFS.

	Overall survival			PFS		
		
Factors	RR	95% CI	*P*-value	RR	95% CI	*P*-value
Age ≥60 vs. <60 years	1.144	0.908-1.427	0.248	1.114	0.898–1.369	0.319
CR vs. other	0.957	0.687–1.274	0.776	0.941	0.693–1.230	0.679
Extra-nodal sites ≥2 vs. <2	1.412	0.837–2.111	0.176	1.107	0.612–1.713	0.699
IPI ≥2 vs. <2	1.388	1.072–1.664	<0.0103	1.307	1.064–1.600	<0.011
LDH >normal	1.321	1.052–1.646	<0.017	1.291	1.043–1.586	<0.0197
PS ≥2 vs. <2	1.615	1.004–2.353	<0.0482	1.311	0.777–1.955	0.277
Stage III/IV vs. I/II	1.069	0.853–1.359	0.567	1.052	0.853–1.314	0.641
TILC (continuous variable)	0.989	0.980–0.997	<0.01	0.992	0.984–0.999	<0.0492
TILC ≥55 d vs. <55 d	0.685	0.542–0.857	<0.0008	0.733	0.592–0.900	<0.0028

CR, complete remission; IPI, international prognostic index; LDH, lactate dehydrogenase; PS, performance status; RR, relative risk; TILC, time interval from last chemotherapy.

**Table IV:** Multivariate analysis for overall and PFS.

	Overall survival			Progression-free survival		
		
Factors	RR	95% CI	*P*-value	RR	95% CI	*P*-value
IPI ≥2 vs. <2	1.192	0.917–1.539	0.187	1.279	1.041–1.567	<0.0195
LDH >normal	1.162	0.887–1.509	0.273			
PS ≥2 vs. <2	1.217	0.740–1.839	0.41			
TILC ≥55 d vs. <55 d	0.703	0.555–0.880	<0.002	0.746	0.602–0.916	<0.0195

IPI, international prognostic index; LDH, lactate dehydrogenase; PS, performance status; RR, relative risk; TILC, time interval from last chemotherapy.

## Discussion

The best timing to undergo ASCT has been reported when patients have minimal residual disease or first CR and in patients who have not received multiple prior chemotherapy treatments (Phillip *et al*, 1995; Kiss *et al*, 2005). To our knowledge, no study has looked at the best timing to proceed with ASCT based on the immune status of the host. We have reported that A-ALC is a prognostic factor for survival post-ASCT in NHL (Porrata *et al*, 2004). An A-ALC is dependent on PA-ALC, as a surrogate marker of immune status of the host at the time of aphaeresis (Porrata *et al*, 2004). It was hypothesised that TILC, as a marker of immune recovery after the myelosuppressive effect of chemotherapy, affects PA-ALC. Therefore, we set out to identify if TILC influences PA-ALC and clinical outcomes of NHL patients treated with ASCT

The stratification of our cohort group based on TILC ≥55 d was balanced for all patients’ characteristics and prognostic factors. The time to proceed to ASCT from last chemotherapy was based on the availability of the aphaeresis and bone marrow transplantation unit to accommodate the patient. The reason for the different conditioning regimens is based on our evolving clinical practice, as cyclophosphamide/TBI was originally used at the start of our transplant programme, followed by BEAC, and BEAM is our current standard conditioning regimen for NHL. A confounding factor affecting TILC is the clinical status of the patient prior to transplantation. Patients in CR or PR and better controlled disease could have waited longer compared with patients with a history of rapid relapses and higher tumour growth rates that might be moved to have an ASCT and stem cell collection sooner and inherited had a worse prognosis. However, both groups were balanced for the clinical status prior to transplantation and only 5% of patients were in 1st or 2nd relapse and refractory disease. No independent correlation was identified between PA-ALC and patients’ baseline characteristics and prognostic factors except for TILC in our cohort of NHL patients. Herein, we present data supporting the observation that PA-ALC is directly influence on the TILC with direct impact on clinical outcomes.

Our study shows a strong correlation between the TILC and PA-ALC at the time of aphaeresis for ASCT in NHL. Patients with a TILC ≥55 d achieved a higher PA-ALC compared with those who did not. The higher PA-ALC translated with a higher A-ALC collection as a strong correlation between PA-ALC and A-ALC was again observed in this study. Consequently, the higher A-ALC collected because of the higher PA-ALC in the TILC ≥55 d translated into a higher ALC-15 recovery and superior OS and PFS in this group compared with patients with a TILC <55 d. Others (Mackall *et al*, 1995) have reported that immunological recovery (including ALC) is delayed after chemotherapy and that ALC increases over time. However, no consideration has been given to the impact of time to immune recovery after chemotherapy and survival, and specifically in the ASCT setting.

The discovery that the autograft immune content directly affects immune recovery and survival post-ASCT is changing the concept of the autograft from a collection of stem cells for haematological engraftment post-ASCT to an adoptive immunotherapeutic strategy to improve immune recovery and survival post-ASCT, similar to the concept of donor lymphocyte infusion (Porrata & Markovic, 2004). However, in this case, the immunotherapeutic manoeuvre would be an autologous lymphocyte infusion (Porrata *et al*, 2006). Recently, Dean *et al* (2005) reported that higher dendritic cells autograft content and recovery post-ASCT translated in superior survival in patients with diffuse large cell lymphoma. We have demonstrated that patients that collected and were infused with higher A-ALC resulted in better survival compared with those who did not (Porrata *et al*, 2004). Rosinski *et al* (2005) reported that premobilisation T-cell status was associated with survival after ASCT. All these findings support the concept that an immunocompetent host at the time of aphaeresis collection translates into better survival after ASCT.

However, not many patients have the luxury of waiting for 2 months to allow for their immune system to recover before proceeding with ASCT. Therefore, autologous immunological graft engineering is warranted (Porrata *et al*, 2005). Methods to engineer an autologous graft-*versus*-tumor effect in ASCT include: *ex vivo* expansion and stimulation of autologous T cells and natural killer cells (Lapport *et al*, 2003; Rapaport *et al*, 2005); the development of autologous lymphocyte mobilisation regimens (i.e. cytokines) to augment the number of immune effector cells into PA-ALC for collection (Sosman *et al*, 2001); and autograft lymphocytes harvesting strategies, such as maximising the capacity of the aphaeresis machine to collect more immune effector cells (Katipamula *et al*, 2006). We hope that these data will support further investigation aimed at maximising patients’ immunological status at the time of ASCT.

## References

[b1] Cheson BD, Horning SJ, Coiffier B, Shipp MA, Fisher RI, Connors JM, Lister TA, Vose J, Grillo-Lopez A, Hagennbeek A, Cabanillas F, Kippensten D, Hiddemann W, Castellino R, Harris NL, Armitage JO, Carter W, Hoppe R, Canellos GP (1999). Report of an international workshop to standardize response criteria for non-Hodgkin's lymphoma. Journal of Clinical Oncology.

[b2] Cox DR (1972). Regression models and life-tables. Journal of the Royal Statistical Society Series B – Statistical Methodology.

[b3] Crowley JJ, McCoy J (1993). Survival trees by goodness of split. Journal of the American Statistical Association.

[b4] Dean R, Masci P, Pohlman B, Andersen S, Serafino S, Sobecks R, Kuczkowski E, Curtis J, Maciejewski J, Rybicki L, Kalaycio M, His E, Theil K, Bolwell BJ (2005). Dendritic cells in autologous hematopoietic stem cell transplantation for diffuse large B-cell lymphoma: graft content and post transplant recovery predict survival. Bone Marrow Transplantation.

[b5] Kaplan E, Meier P (1958). Nonparametric estimation from incomplete observations. Journal of the American Statistical Association.

[b6] Katipamula R, Porrata LF, Gastineau DA, Markovic SN, Moore SB, Greiner C, Burgslater EA, Padley D, Winters JL (2006). Apheresis instrument settings influence infused absolute lymphocyte count affecting survival following autologous peripheral hematopoietic stem cell transplantation in non-Hodgkin's lymphoma: the need to optimize instrument settings and define a lymphocyte collection target. Bone Marrow Transplantation.

[b7] Kiss TL, Mollee P, Lazarus HM, Lipton JH (2005). Stem cell transplantation for mantle cell lymphoma: if, when and how?. Bone Marrow Transplantation.

[b8] Lapport GG, Levine BL, Sadtmauer EA, Schuster SJ, Luger SI, Grupp S, Bunin N, Strobl FJ, Cotte J, Zheng ZH, Gregson B, Rivers P, Vonderheide RH, Liebowitz DN, Porter DL, June CH (2003). Adoptive transfer of costimulated T cells induces lymphocytosis in patients with relapsed/refractory non-Hodgkin lymphoma following CD34+ selected hematopoietic cell transplantation. Blood.

[b9] Mackall CL, Fleisher TA, Brown MR, Andrich MP, Chen CC, Feuerstein IM, Horowitz ME, Magrath IT, Shad AT, Steinberg SM, Wexler LH, Gress RE (1995). Age, thymopoiesis, and CD4+ T-lymphocyte regeneration after intense chemotherapy. New England Journal of Medicine.

[b10] McShane LM, Altman DG, Sauerbrei W, Taube SE, Gion M, Clark GM, for the Statistics Subcommittee of the NCI-EORTC Working Group on Cancer Diagnostics. (2005). Reporting recommendations for tumor MARKer prognostic studies (REMARK). European J Cancer.

[b11] Phillip T, Guglielmi C, Hagenbeek A, Somers R, Van der Lelie H, Bron D, Sonneveld P, Gisselbrecht C, Cahn JY, Harousseau JL, Coiffier B, Biron P, Mandelli F, Chauvin F (1995). Autologous bone marrow transplantation as compared with salvage chemotherapy in relapse of chemotherapy-sensitive non-Hodgkin's lymphoma. New England Journal of Medicine.

[b12] Porrata LF, Markovic SN (2004). Timely reconstitution of immune competence affects clinical outcome following autologous stem cell transplantation. Clinical and Experimental Medicine.

[b13] Porrata LF, Litzow MR, Inwards DJ, Gastineau DA, Moore SB, Pineda AA, Bundy KL, Padley DJ, Persky D, Ansell SM, Micallef IN, Markovic SN (2004). Infused peripheral blood autograft absolute lymphocyte count correlates with day 15 absolute lymphocyte count and clinical outcome after autologous peripheral hematopoietic stem cell transplantation in non-Hodgkin's lymphoma. Bone Marrow Transplantation.

[b14] Porrata LF, Litzow MR, Markovic SN (2005). Graft engineering for autologous stem cell transplantation. Gene Therapy and Molecular Biology.

[b15] Porrata LF, Litzow MR, Gastineau DA, Markovic SN (2006). Autologous lymphocyte infusion as an immunotherapeutic strategy in autologous stem cell transplantation. Research Advances in Cancer.

[b16] Rapaport AP, Stadtmauer EA, Aqui N, Badros A, Cotte J, Chrisley L, Veloso E, Zheng ZH, Westphal S, Mair R, Chi N, Ratterree B, Pochran MF, Natt S, Hinkle J, Sickles C, Sohal A, Ruehle K, Lynch C, Zhang L, Porter DL, Luger S, Guo CF, Fang HB, Blackwelder W, Hankey K, Mann D, Edelman R, Frasch C, Levine BL, Cross A, June CH (2005). Restoration of immunity in lymphopenic individuals with cancer by vaccination and adoptive T-cell transfer. Nature Medicine.

[b17] Rosinski SL, McNiece IK, Shpall EJ, Clough N, Russell P, Blunk B, Nieto Y (2005). Prognostic analysis of pre-transplant peripheral T-cell levels in patients receiving an autologous hematopoietic progenitor-cell transplant. Bone Marrow Transplantation.

[b18] Sosman JA, Stiff P, Moss SM, Sorokin P, Martone B, Bayer R, van Besien K, Devine S, Stock W, Peace D, Chen Y, Long C, Gustin D, Viana M, Hoffman R (2001). Pilot trial of interleukin-2 with granulocyte colony-stimulating factor for the mobilization of progenitor cells in advanced breast cancer patients undergoing high-dose chemotherapy: expansion of immune effectors within the stem-cell graft and post-stem-cell infusion. Journal of Clinical Oncology.

